# Characterization of the catalytic center of the Ebola virus L polymerase

**DOI:** 10.1371/journal.pntd.0005996

**Published:** 2017-10-09

**Authors:** Marie Luisa Schmidt, Thomas Hoenen

**Affiliations:** Institute for Molecular Virology and Cell Biology, Friedrich-Loeffler-Institut, Greifswald–Insel Riems, Germany; University of Texas Medical Branch, UNITED STATES

## Abstract

**Background:**

Ebola virus (EBOV) causes a severe hemorrhagic fever in humans and non-human primates. While no licensed therapeutics are available, recently there has been tremendous progress in developing antivirals. Targeting the ribonucleoprotein complex (RNP) proteins, which facilitate genome replication and transcription, and particularly the polymerase L, is a promising antiviral approach since these processes are essential for the virus life cycle. However, until now little is known about L in terms of its structure and function, and in particular the catalytic center of the RNA-dependent RNA polymerase (RdRp) of L, which is one of the most promising molecular targets, has never been experimentally characterized.

**Methodology/Principal findings:**

Using multiple sequence alignments with other negative sense single-stranded RNA viruses we identified the putative catalytic center of the EBOV RdRp. An L protein with mutations in this center was then generated and characterized using various life cycle modelling systems. These systems are based on minigenomes, i.e. miniature versions of the viral genome, in which the viral genes are exchanged against a reporter gene. When such minigenomes are coexpressed with RNP proteins in mammalian cells, the RNP proteins recognize them as authentic templates for replication and transcription, resulting in reporter activity reflecting these processes. Replication-competent minigenome systems indicated that our L catalytic domain mutant was impaired in genome replication and/or transcription, and by using replication-deficient minigenome systems, as well as a novel RT-qPCR-based genome replication assay, we showed that it indeed no longer supported either of these processes. However, it still showed similar expression to wild-type L, and retained its ability to be incorporated into inclusion bodies, which are the sites of EBOV genome replication.

**Conclusions/Significance:**

We have experimentally defined the catalytic center of the EBOV RdRp, and thus a promising antiviral target regulating an essential aspect of the EBOV life cycle.

## Author summary

Ebola viruses cause severe hemorrhagic fevers, and were responsible for the devastating Ebola virus disease epidemic in West Africa from 2013 to 2016. While a number of experimental therapeutics against these viruses target the viral polymerase, there are still significant gaps in our knowledge regarding this essential viral protein. In particular, until now no experimental evidence has been provided identifying the catalytic center of the viral RNA-dependent RNA polymerase, which is absolutely essential for the virus life cycle due to its role in replicating and transcribing the viral negative-sense RNA genome. Based on a comparison to related negative-sense RNA viruses from other virus families we identified a putative catalytic center within the Ebola virus polymerase, and provide the experimental evidence that the Ebola virus polymerase indeed utilizes a classical GDNQ motif for both genome replication and transcription. This finding not only increases our knowledge regarding the molecular biology of Ebola viruses, but also defines a molecular target for the development of antivirals against this deadly virus.

## Introduction

Ebola virus (EBOV) is a member of the genus *Ebolavirus* in the family of *Filoviridae*, and the causative agent of a severe hemorrhagic fever called Ebola virus disease (EVD) with case fatality rates of up to 90% [1]. While outbreaks are usually of comparatively small scale, the recent EVD epidemic in West Africa involved more than 28,000 cases with more than 11,000 deaths [2], highlighting the urgent need for effective countermeasures against this virus. Significant progress has been made in recent years on the development of such countermeasures, with experimental vaccines showing promise in phase III clinical trials [3]. Similarly, a number of experimental therapeutics are under development, many of which target the viral polymerase L (reviewed in [4,5]). This viral protein acts in concert with the other viral ribonucleoprotein complex (RNP) proteins, the nucleoprotein NP, the polymerase cofactor VP35, and the transcriptional activator VP30, to facilitate replication of the negative sense RNA genome of EBOV, as well as its transcription into viral mRNAs [6].

Despite its central role in the virus life cycle, relatively little is known about the L protein both in terms of its structure and in terms of functional details, which might in part be due to its large size and the fact that no specific antibodies are available, making biochemical studies of this protein challenging. Thus, much of what we know about L has been elucidated using reverse genetics-based life cycle modelling systems [7]. The most basic of these systems is the minigenome system [6]. Here a miniature version of the viral genome (a so-called minigenome), in which all viral open reading frames have been removed and replaced by a reporter gene, but in which the non-coding terminal leader and trailer regions are retained, is expressed together with the RNP proteins in mammalian cells. These RNP proteins recognize the minigenome as an authentic viral template based on its leader and trailer regions, and replicate and transcribe it resulting in reporter activity levels that mirror these steps in the viral life cycle. As a modification of this classical monocistronic minigenome system, a so-called replication-deficient minigenome system has also recently been developed, which utilizes a minigenome with a deletion in the antigenomic replication promotor [8]. This system allows genome transcription to be investigated in isolation, something that is not possible in the classical system, where reporter activity is always the product of both transcription and genome replication (which amplifies the number of genomic templates available for transcription). Further, with the tetracistronic transcription and replication-competent virus-like particle (trVLP) system another life-cycle modelling system has been developed. This system utilizes a minigenome that encodes not only a reporter, but also the viral proteins VP40, GP_1,2_, and VP24, which are responsible for virus particle morphogenesis and budding, entry and fusion, and proper nucleocapsid assembly, respectively [9,10]. In this system minigenome replication and transcription in so-called producer (p0) cells not only leads to reporter activity, but also to the formation of trVLPs, which package minigenomes-containing nucleocapsid-like structures and can infect target (p1) cells. Using this system it is possible to model virtually the complete virus life cycle outside of a high containment laboratory. In all such minigenome-based systems an important consideration is to control for effects on plasmid-driven gene expression. This is usually done by including a control plasmid that encodes for another reporter, e.g. Firefly luciferase. This allows these cell-based assays to be normalized for well-to-well differences in transfection efficacy, cell density, or experimental effects, e.g. differences in cytotoxicity of tested drugs. Similarly, the extent to which virus RNP complex-specific genome replication and transcription are occurring is typically assessed by omitting the polymerase L.

In the past, minigenome systems have been used to demonstrate the role of L in replication and transcription [6], as well as to investigate the functional interactions of L with VP35 [11]. Indeed, this interaction has been shown to be crucial for genome replication and/or transcription, and a further study revealed an additional interaction of L with VP30 [12]. In that same study, we also identified a flexible linker region in L that is tolerant to insertions, and by subsequently fusing mCherry into this region we were able to characterize the intracellular fate of this protein, and show that it localizes in so-called inclusion bodies, which are formed in virus-infected cells and act as sites of genome replication [13]. However, fundamental aspects of L have still not been investigated, and for example the catalytic center of the RNA-dependent RNA-polymerase (RdRp) has not been experimentally determined, even though this information would be of great importance for drug development efforts [5]. In contrast, for other negative sense RNA viruses this catalytic center is well defined and involves a GDNQ motif, which based on bioinformatics analysis has been proposed to also be present and functional in the filovirus polymerase [14,15]. This motif represents a variant of the GDD motif found in RdRps of other viruses [16], and sits in a deep channel of the polymerase [17], where it complexes two metal ions essential for polymerase function [18]. Here, we provide the first experimental evidence that this motif is indeed essential for both virus genome replication and transcription, providing further insight into the molecular biology of filoviruses, and defining an important molecular target for the development of antiviral compounds against this deadly virus.

## Methods

### Multiple sequence alignment

Filovirus reference sequences (NC_014373, NC_016144, NC_001608, NC_004161, NC_006432, NC_014372, NC_002549) [19] as well as reference sequences for RSV (NC_001803) and VSV (NC_001560) were obtained from GenBank. Sequences were imported into Geneious v10.0.9 (Biomatters), the L open reading frames were translated, and a multiple sequence alignment was performed using the ClustalW algorithm and the BLOSUM substitution matrix series. For calculating similarities in the pairwise distance analysis, amino acids were deemed similar if exchanges between those amino acids reached or exceeded a threshold of 0 in a BLOSUM62 matrix.

### Cells

HEK 293T (human embryonic kidney; Collection of Cell Lines in Veterinary Medicine CCLV-RIE 1018) cells were maintained in Dulbecco´s modified Eagle’s minimum essential medium (DMEM; ThermoFisher Scientific) supplemented with 10% fetal bovine serum (FBS; Biochrom), and 100 U/ml penicillin and 100 μg/ml streptomycin (P/S; ThermoFisher Scientific). Huh7 cells (human hepatoma cells; Collection of Cell Lines in Veterinary Medicine CCLV-RIE 1079) were cultured in a 1:1 mix of Ham’s nutrient mixture F-12 (ThermoFisher Scientific) and Iscove's modified Dulbecco's medium (IMDM; ThermoFisher Scientific) supplemented with 10% FBS, 100 U/ml penicillin and 100 μg/ml streptomycin. All cells were grown at 37°C with 5% CO_2_.

### Plasmids

pCAGGS expression plasmids for NP, VP35, VP30, L, L-mCherry, T7, firefly luciferase, Tim1, and replication-competent and -deficient monocistronic minigenomes as well as the tetracistronic minigenome have been previously described [8,13]. A GFP-expressing minigenome was generated by deleting the luciferase open reading frame (ORF) from a luciferase-expressing minigenome and replacing it with the eGFP ORF using conventional PCR techniques and cloning with type IIS restriction enzymes. Primers and details of the cloning strategy are available upon request. Mutation of the EBOV-L gene (specifically A13805C, A13807G, A13808C, A13811C, with all positions relative to the full length EBOV genome) was performed by a combination of conventional and overlap extension PCR methods. To this end, first two separate touchdown PCRs using IProof polymerase (Biorad) and pCAGGS-L as template with the primers 5’-CCCGGGGCGGCCGCAAATG-3’ and 5’-CACCCATCACAGCTGAGCGTAACTTAAAAC as well as 5’-GTTTTAAGTTACGCTCAGCTGTGATGGGTGCCGCTGCGTGCATTACTGTTTTATC-3’ and 5’-GTTTGCCGAGTGTTAACTGTCCAAGG-3’ were performed. PCR-products were digested with DpnI (New England Biolabs, NEB), and a second PCR was performed using the two PCR products as template, and the primers 5’-CCCGGGGCGGCCGCAAATG-3’ and 5’-GTTTGCCGAGTGTTAACTGTCCAAGG-3’. The final PCR-product was cloned via NotI (NEB) and HpaI (NEB) into pCAGGS-L. To generate the mutant L fused to mCherry the region of pCAGGS-L_mut_ with the mutation was subcloned into pCAGGS-L-mCherry using the restriction enzymes HpaI and NotI. All plasmids were sequence confirmed by Sanger-sequencing.

### Minigenome assays

Minigenome assays were performed as previously described [8], with slight modifications. HEK 293T cells were seeded into 12 well plates, and transfected at a confluency of about 50% using 3 μl Transit LT1 (Mirus) per μg DNA with expression plasmids encoding NP (62.5 ng), VP35 (62.5 ng), VP30 (37.5 ng), T7-polymerase (125 ng), firefly luciferase (12.5 ng), L or L_mut_ (500 ng) or an equivalent amount of empty vector in the -L control, and a replication-competent minigenome (transcription and replication assay) or a replication-deficient monocistronic minigenome (transcription assay) (125 ng) with Renilla luciferase as the reporter. At 24 hours post transfection (p.t.), medium was exchanged against 2 ml of DMEM supplemented with 5% FBS and P/S, and after 48 hours p.t. luciferase activity was measured. To this end the supernatant was removed from the cells, 200 μl 1x Lysis Juice (PJK) was added to the cells, and after 10 minutes incubation at room temperature the lysate was removed and cell debris spun down 3 minutes at 10,000 x g. Then 40 μl clarified lysate was added to 40 μl Beetle Juice (PJK) or 40 μl Renilla Glo Juice (PJK) in black opaque 96-well plates, and luminescence was measured using an Infinite F200 PRO (Tecan) multimode reader with an integration time of 1 sec. Renilla luciferase activities were normalized to Firefly luciferase activities.

### Replication assay

To assess genome replication in isolation, a modified transcription and replication-competent virus-like particle (trVLP) assay [10] was combined with a newly developed RT-qPCR. HEK 293T producer cells (p0) were transfected with expression plasmids for NP, VP35, VP30, the T7-polymerase, L, and a tetracistronic minigenome to generate trVLPs for infection of HEK 293T target cells (p1) as previously described [10]. Target p1 cells in 12-well format were pre-transfected with pCAGGS-NP (62.5 ng), pCAGGS-VP35 (62.5 ng), pCAGGS-Tim1 (125 ng), as well as pCAGGS-L or pCAGGS-L_mut_ (500 ng). 24 hours p.t. these p1 cells were infected with 1.5 ml of clarified (5 minutes at 800 x g and room temperature), pooled p0 supernatant containing trVLPs. In order to do so, trVLP-containing supernatant was added to p1 cells, and the cells were centrifuged for 10 minutes at 1,000 x g, and then incubated at 37°C for 1 hour. After that, the inoculum was exchanged against 2 ml DMEM with 5% FBS and P/S. No VP30 was expressed in these p1 cells, so that only genome replication but not transcription could take place [6]. 48 hours post infection cells were lysed and RNA was isolated using the NucleoSpin RNA kit (Macherey-Nagel) following the manufacturer’s instructions. An additional DNA digestion step was performed using the turbo DNA-free kit (ThermoFisher Scientific) after RNA purification following the manufacturer’s instructions. RNA samples were then quantified by real-time RT-qPCR using the AgPath-ID One Step RT-PCR kit (Applied Biosystems), with EBOV_IGR: 5’-6FAM-CAATAGCCAATACCAAACACCTCCTCCACAGCTTG-BHQ1-3’ as probe, and the primers EBOV_IGR-fwd2 5’-TCACAATCTACCTCTTGAAACAAGAAC-3’ and EBOV_IGR-rev2 5’-CATGACTTACTAATGATCTCTTAAAATATTAAG-3’ in 3 technical replicates, the results of which were averaged. To allow absolute quantification of copy numbers an RNA standard was prepared by *in vitro* transcription of the tetracistronic minigenome using the TranscriptAid T7 High Yield Transcription kit (ThermoFisher Scientific) following the manufacturer’s instructions, and quantified using a P-class P330 nanophotometer (IMPLEN). 10^5^, 10^7^, and 10^9^ RNA copies were used as standards in the real-time RT-qPCR.

### Analysis of L expression

For western blot analysis Huh7 cells were seeded into 12 well plates and transfected as described above for the replication and transcription minigenome assay, with pCAGGS-L-mCherry, pCAGGS-L_mut_-mCherry, or empty vector (-L control) in place of pCAGGS-L. After 24 hours p.t. the medium was changed to 1 ml medium supplemented with 5% FBS and P/S. The cells were lysed after 48 hours p. t. in 1x SDS sample buffer (10% glycerol, 5% 2-mercaptoethanol, 2% SDS, 37.5 mM Tris-HCl, 2.5 μg/ml bromphenol blue), incubated at 95°C for 5 minutes, and lysates were analyzed by SDS-PAGE and western blotting as previously described [20] using anti-mCherry (Biozol: 1:1000) and anti-actin (Sigma-Aldrich: 1:2000) primary antibodies and a peroxidase conjugated goat-anti-mouse secondary antibody (Diavona: 1:10000).

For localization studies, Huh7 cells in 4-well μ-slides (Ibidi) were transfected with the same plasmids as for the western blot analysis but using half the amount of plasmid per well and a GFP-expressing minigenome. Additionally, 125 ng pmTurquoise2-H2A, which was a gift from Dorus Gadella (Addgene plasmid # 36207)[21], was cotransfected in order to label cell nuclei. Cells were visualized by spinning disc live cell microscopy using a Leica DMi8 with a Yokogawa CSU-W1 confocal scanning head, an Andor iXon Ultra 888 EMCCD camera, and 445 nm, 488 nm, and 561 nm laser lines. All images were taken using identical laser and camera settings for each wavelength.

### Statistical analysis

Paired two-tailed t-tests were performed using the GraphPad online QuickCalc (https://www.graphpad.com).

## Results

### L contains a classical GDNQ polymerase motif

**Fig 1:**
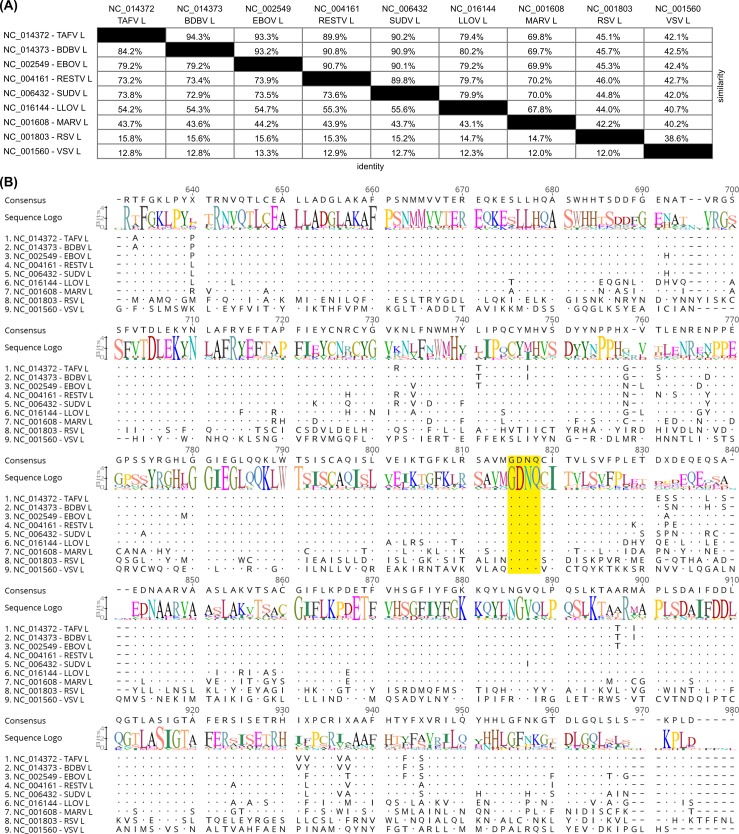
Multiple sequence alignment of filovirus, RSV, and VSV polymerases. **(A) Pairwise distance of aligned sequences.** The percentage of identical (bottom left) or similar (top right) amino acids is shown. **(B) Positions 631 to 980 of the multiple sequence alignment.** Dots indicate amino acids identical to the consensus sequence. The conserved GDNQ motif is highlighted in yellow.

For many negative sense RNA polymerases the catalytic center of their RdRp has been well defined, and incorporates a GDNQ motif [4]. Therefore, in order to identify the putative catalytic center of the EBOV polymerase, a multiple sequence alignment was performed between L open reading frames (ORFs) obtained from reference sequences for all filoviruses (the ebolaviruses EBOV, Sudan virus (SUDV), Bundibugyo virus (BDBV), Reston virus (RESTV), and Taï Forest virus (TAVF), the marburgvirus Marburg virus (MARV), and the cuevavirus Lloviu virus (LLOV)) [19], as well as Respiratory Syncytial Virus (RSV) and Vesicular Stomatitis Virus (VSV). The alignment showed a very high degree of conservation among the filovirus polymerases (Fig 1A), with the ebolavirus polymerases showing 90 to 94% sequence similarity and 73 to 84% sequence identity to each other, 79 to 80% similarity and 54 to 56% identity to LLOV L, and 70% similarity and 44% identity to MARV L. LLOV L showed a higher similarity and identity to the ebolaviruses polymerases (79 to 80% similarity and 54 to 56% identity) than to MARV L (68% similarity and 43% identity), consistent with previous reports regarding the phylogenetic relationships between these genera [22]. As expected, the similarity to RSV and VSV was much lower, with 45 to 46% similarity and 15 to 16% identity between ebolaviruses and RSV, and 42 to 43% similarity and 13% identity between ebolaviruses and VSV. Nevertheless, a conserved GDNQ motif could easily be identified (Fig 1B) at positions 741–744 of the EBOV polymerase sequence (position 815–818 in the multiple sequence alignment), and was identical in all analyzed sequences.

### Abrogating the GDNQ motif does not affect L expression or localization

**Fig 2:**
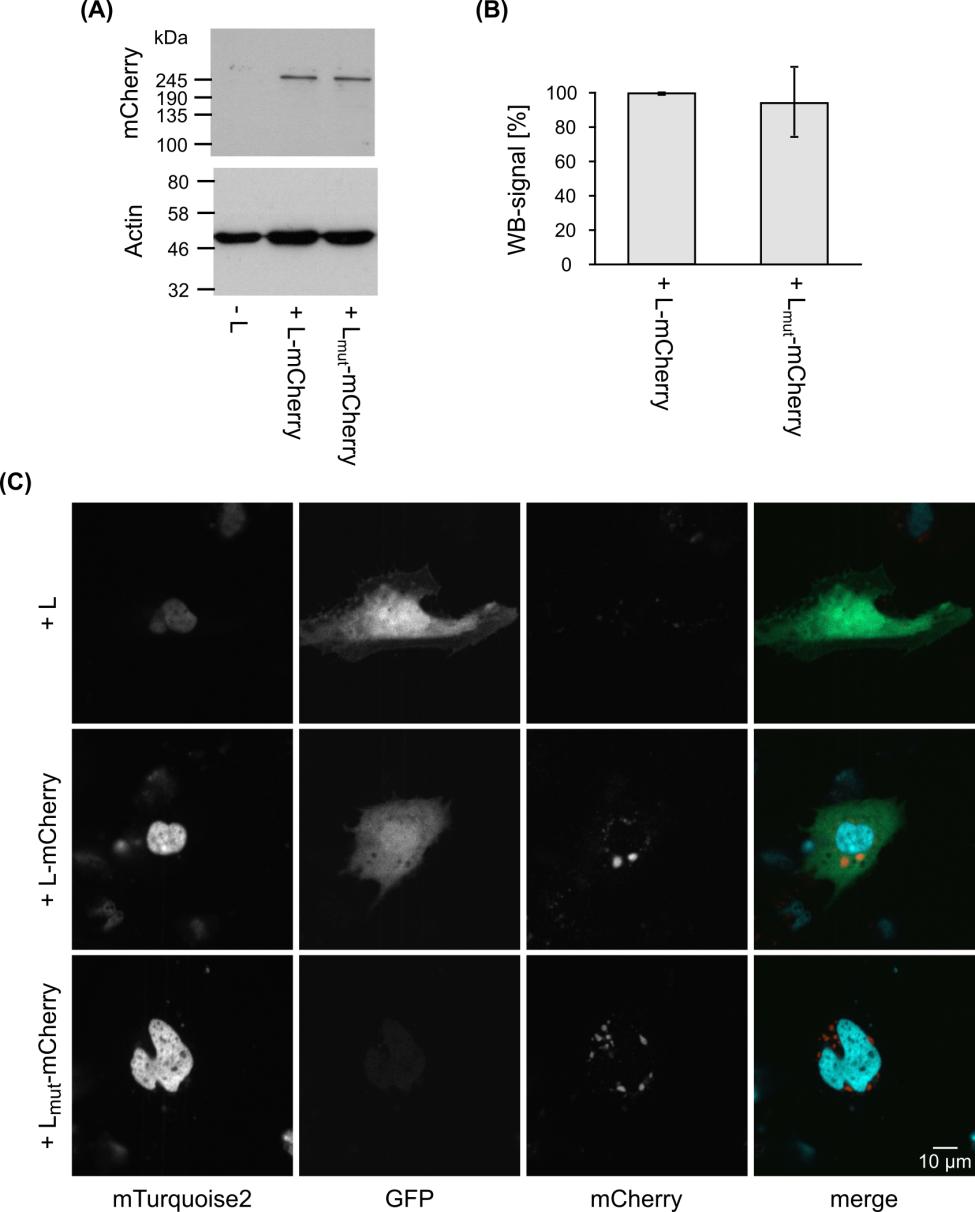
Effect of mutation of the GDNQ motif on expression and intracellular localization of EBOV L. **(A) Western blot of 293T cell lysates transfected with L-mCherry or L**_**mut**_**-mCherry.** Blots were cut in half, and stained with monoclonal antibodies against mCherry or actin. **(B) Quantification of L-mCherry expression.** Results from western blotting were quantified, and the expression level of wild-type L set to 100%. Means and standard deviations of 4 biological replicates from 3 independent experiments are shown. **(C) Intracellular localization of L-mCherry or L**_**mut**_**-mCherry.** Cells were transfected with expression plasmids for all RNP proteins (including either wild-type L, L with a fluorescent mCherry tag (L-mCherry) or a mutated version of L-mCherry without the GDNQ motif (L_mut_-mCherry)), a GFP-expressing replication-competent minigenome, T7-polymerase to facilitate initial transcription of minigenome vRNA, and pmTurquoise2-H2A to label cell nuclei. Live cells were visualized after 48 hours by spinning disc confocal microscopy. The white bar indicates 10 μm.

After having identified a putative catalytic center within the EBOV L, we generated expression plasmids in which this motif was mutated by substitution of the DNQ sequence to 3 alanine residues. In order to assess whether these mutations affected expression of the protein, we performed western blot analysis after transient expression in 293T cells. Since no L-specific antibodies are available, we instead used L versions in which the fluorescent tag mCherry had been inserted into a flexible linker region, which we have previously shown tolerates insertions well without dramatically impacting protein function [13]. By western blotting we did not observe any significant differences (p = 0.708) in expression level (Fig 2A and 2B) between L with an intact GDNQ motif and a mutated GAAA motif.

In order to further assess whether there were changes to the intracellular localization of mutated L, we again used mCherry-tagged variants of L in combination with an eGFP-expressing minigenome. As expected based on previous studies, L-mCherry with an intact GDNQ motif localized into punctate structures that most likely represent early inclusion bodies (Fig 2C) [13]. Similar structures were also observed in the presence of L-mCherry with an abrogated putative catalytic domain. However, unlike the situation with untagged wild-type L, or L-mCherry with an intact GDNQ motif, we did not observe any GFP reporter activity with the L GAAA mutant, strongly suggesting a lack of activity of this mutant in transcription and/or replication.

### Abrogating the GDNQ motif completely inhibits viral genome replication and transcription

**Fig 3:**
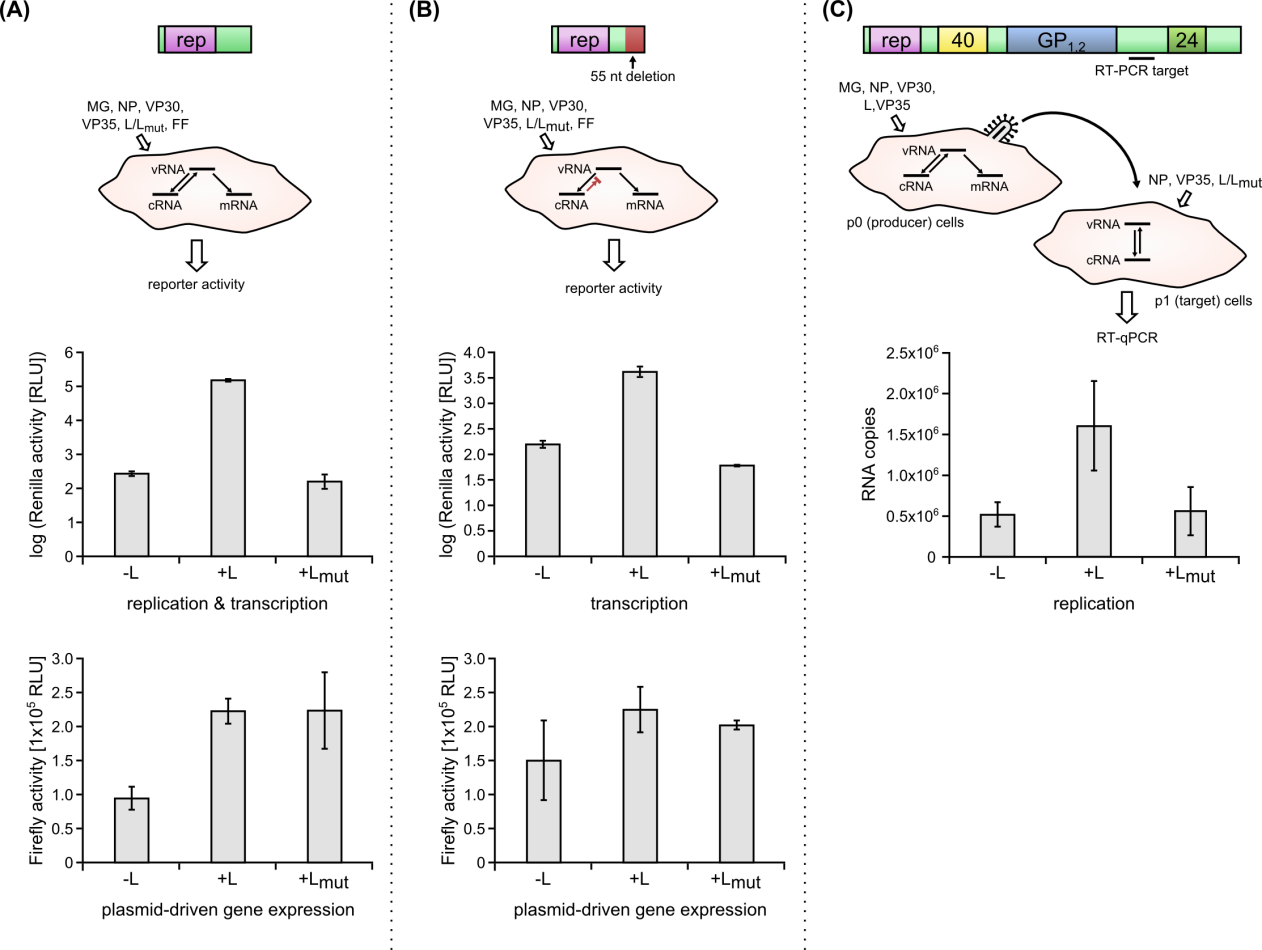
Functional characterization of the GDNQ motif in EBOV L on viral transcription and replication. **(A) Effects of the GDNQ mutation in L on genome replication and/or transcription.** A classical minigenome (MG) assay using a Renilla luciferase reporter (rep) was performed with wild-type L (L) or the mutated L (L_mut_). In this assay reporter activity (top graph, shown in relative light units (RLU) on a log scale) reflects genome replication and transcription in combination, but doesn’t distinguish between effects on one or both of these processes. In addition, plasmid-based gene expression from a Firefly (FF) luciferase plasmid was measured as a control and is shown in the bottom graph. Means and standard deviations of 3 biological replicates from 3 independent experiments are shown. **(B) Effects of the GDNQ mutation in L on genome transcription.** A replication-deficient minigenome assay was performed with wild-type and mutated L. In this assay, cRNAs cannot be copied back into vRNAs, abolishing replication, and thus reporter levels become independent of replication, which normally amplifies the available number of vRNA templates for reporter mRNA transcription. Therefore, Renilla luciferase reporter activity in this system reflects genome transcription alone. Again, plasmid-based gene expression from a Firefly (FF) luciferase plasmid was measured as a control and is shown in the bottom graph. Means and standard deviations of 3 biological replicates from 3 independent experiments are shown. **(C) Effects of the GDNQ mutation in L on genome replication.** p1 cells were pretransfected with NP, VP35, and L or L_mut_, and then infected with trVLPs containing a tetracistronic minigenome. Replication of this minigenome in p1 cells was measured by RT-qPCR targeting the intergenic region located between the GP_1,2_ and VP24 ORFs on the minigenome. Means and standard deviations of 4 biological replicates from 2 independent experiments are shown.

Given the strong impact of the GDNQ motif on reporter expression in context of the GFP-encoding minigenome, we next sought to quantify this impact using a Renilla luciferase-expressing minigenome, which allows easier quantification and more sensitive detection of reporter activity. To this end, we first performed classical minigenome assays, which measure both genome replication and transcription at the same time, but do not distinguish between these two steps. In this system, when using the L mutant with the abrogated GDNQ motif we observed a complete loss of reporter activity (i.e. >1000 fold reduction) with signals being reduced down to the background levels observed also in the complete absence of L, clearly indicating that this motif is absolutely essential for EBOV genome replication, transcription, or both of these processes (Fig 3A) (-L vs. +L: p = 0.001; +L vs. +L_mut_: p = 0.003). When looking at the control Firefly luciferase, it became apparent that there was significantly less Firefly signal in the -L control than in the +L sample (p = 0.019). However, this difference was only about 2.4 fold, and did not contribute appreciably to the difference in the Renilla reporter signal, which was several orders of magnitude larger (i.e. 1433 fold).

In order to distinguish whether genome replication or transcription or both processes were impaired by mutation of the GDNQ motif, we next used a replication-deficient minigenome [8]. In these experiments absolute reporter levels were considerably lower, also in the positive control using wild-type L, compared to the reporter activity in the replication-competent minigenome (Fig 3B), reflecting the strong contribution of minigenome replication to overall reporter activity in this system (due to amplification of the vRNA templates available to serve as templates for transcription). However, again reporter activity in the presence of the mutated L was 67 fold lower than in the presence of wild-type L, which represents the background level for the assay (-L vs. +L: p = 0.005; +L vs. +L_mut_: p = 0.002). This clearly indicates that the GDNQ mutation is essential for viral transcription, independent of any contribution from effects on viral replication. Again the Firefly signal appeared lower in the -L control compared to the +L sample; however, in this series of experiments this difference was not statistically significant.

Finally, we also wanted to assess an independent impact of the GDNQ motif on genome replication. To this end, we developed a novel replication assay by combining a tetracistronic transcription and replication competent virus-like particle (trVLP) assay [10] with an RT-qPCR. To this end, a tetracistronic minigenome encoding VP40, GP_1,2_, and VP24, in addition to a reporter, was expressed in p0 producer cells in the presence of the RNP proteins. This resulted in the formation of trVLPs that contain copies of the minigenome encapsidated in nucleocapsid-like structures. These trVLPs were then used to infect p1 target cells, which had been pretransfected with expression-plasmids for NP, VP35, the EBOV adhesion factor Tim1, and either wild-type L, or L with a mutated GDNQ motif. VP30 was intentionally omitted in p1 cells, since this protein has been shown to be an essential transcriptional activator, but not required for replication [6,8]. Total RNA from these p1 cells was harvested 2 days after infection, and subjected to an RT-qPCR assay targeting the intergenic (i.e. non-transcribed) region between the GP gene and the VP24 gene in the minigenome. Again, we saw a significant (-L vs. +L: p = 0.048; +L vs. +L_mut_: p = 0.011) reduction in vRNA/cRNA accumulation down to background levels when using L containing the mutated GDNQ motif, indicating that this motif is also required for genome replication.

## Discussion

The EBOV polymerase is the target for a number of potential antivirals such as favipiravir (T705) [23], BCX4430 [24], GS-5734 [25], and β-D-N^4^-hydroxycytidine [26]. Further, it has been the target for a number of high-throughput drug screens [27–29], which generally exploit minigenome systems to allow rapid and easy modelling of the EBOV life cycle without the need for a high containment facility [30]. However, despite its central role in the virus life cycle, structural and functional data for this protein remains scarce.

When the protein sequences of negative sense RNA virus polymerases of Rhabdo- and Paramyxoviruses were first published [31], it quickly became clear that they share highly conserved regions that we now know to correspond to the RdRp, the polyribonucleotidyltransferase (PRNTase), and the methyltransferase (MTase) domains (reviewed in [32]), and for the Rhabdovirus VSV the structure of these domains has been solved at the atomic level [17]. The same conserved regions have since been tentatively identified based on sequence comparisons in other negative-sense RNA viruses including EBOV and MARV [33,34]. Further, while only limited crystal structure information is available for negative sense RNA virus polymerases, bioinformatics-based structural predictions suggest that the filovirus polymerase has a similar structure than polymerases from viruses for which a structure is known [14,15]. Experimental evidence of such a similar structure and experimental identification of molecular targets within the polymerase can help in rational drug design, as well as provide important insight in the mechanisms of action of compounds targeting L.

This conservation of sequence and structure was the basis for the identification of flexible linker sites that allowed insertion of peptide tags as well as fluorescent proteins into the EBOV polymerase, with little impact on its expression, localization or function [12,13], similar to previous studies involving the polymerases of paramyxoviruses [35,36]. Further, this assumption formed the basis for the multiple sequence alignment used in the present study to search for a putative catalytic center of the EBOV polymerase. Using this alignment, a GDNQ motif in the RdRp was readily identified, consistent with predictions by Cong et al., who have suggested that D742 is a catalytic site in the filovirus polymerase [14]. After mutating the GDNQ motif, our functional results using luciferase-encoding minigenomes showed very clearly that this motif is required for genome replication and/or transcription of EBOV, and that this mutation completely abolishes transcription (based on the results of the replication-deficient minigenome system) and potentially both of these processes, resulting in reporter levels that are identical to samples completely lacking viral polymerase, and corresponding to the background noise of the luminometer (about 10^2^ RLU). Similar results were observed using GFP as a reporter, where in cells expressing L-mCherry with a mutated catalytic domain no GFP signal was observed. This was in contrast to cells expressing wild-type L-mCherry, where a strong GFP signal was readily observed, corresponding to robust minigenome transcription and replication (albeit not in all cells, since in addition to L the other RNP proteins, as well as the minigenome, all have to be expressed in the same cell).

In order to show definitively that genome replication is also abolished, we developed a replication assay by combining RT-qPCR technology and the recently published tetracistronic trVLP system. This approach has the advantage that neither minigenome-encoding plasmid DNA nor initial T7-transcribed and naked minigenome RNA is present in the p1 cells analyzed, since the source of the minigenome in those cells is infecting trVLPs which have packaged minigenome RNA-containing nucleocapsid like-structures [10]. Further, the target of the RT-qPCR is the VP30/VP24 intergenic region. In the tetracistronic minigenome this sequence is located between the GP_1,2_ ORF and the VP24 ORF (in contrast, in the full-length EBOV the GP and VP24 genes are separated by the VP30 gene, so that no native GP/VP24 gene junction exists). This region harbors the longest non-transcribed sequence in the EBOV genome with a length of 144 nt [37]. This approach allowed us to exclude detection of mRNA, rather than cRNA/vRNA, despite the use of a one-step RT-PCR (i.e. instead of a strand-specific two-step RT-PCR to target vRNA specifically). Additionally, we further exclude the erroneous detection of mRNA in this system by omitting expression of the transcriptional activator VP30 in p1 cells, as this protein has been shown to be required for transcription, but not for genome replication [6,8].

While, as with all point mutations, there is always the concern that the introduced mutations might negatively affect protein folding, we believe this not to be the case in this instance for two reasons: First, the mutated mCherry-tagged polymerase is readily recruited into inclusion bodies similar to those observed previously in cells infected with a recombinant EBOV expressing L-mCherry at early time points after infection, indicating that it still has to be able to interact with the other RNP proteins. Secondly, we have previously shown that an interaction with VP35 is required for stable expression of L, and that in the absence of VP35 L cannot be detected in significant amounts by western blotting [13]. The interaction domain between VP35 and L has been mapped to the amino acids 280 and 370, which are located in the RdRp domain of L [11]. Since we do not see any differences in the expression level of our mutated L, we have to conclude that this mutant remains able to interact with VP35, and that, therefore, the RdRp domain which harbors the mutation is not grossly misfolded.

On a technical note, analysis of the Firefly control luciferase in this study showed signals for this reporter that were lower in the -L controls than in the +L samples (regardless of whether L was functional or not). This phenomenon is most likely due to the fact that it is common good practice to include empty vector in samples where plasmids are omitted for experimental reasons (e.g. in -L controls), in order to equalize the transfected plasmid mass. However, given the size of the L expression plasmid (11.6 kB) vs. the empty vector (4.8 kB), this means that in terms of absolute numbers many more empty plasmids than L expression plasmids are transfected, which may lead to a reduction in gene expression from the other co-transfected plasmids. This effect can skew the results of minigenome assays, since reporter luciferase activity values are normalized to these control luciferase values, thus artificially inflating -L control values. While this effect is small compared to the very large dynamic range of EBOV minigenome assays (which in our hands is about 3 log_10_), particularly for high-throughput assays were a large dynamic range is required and this control luciferase is essential to normalize for well-to-well variations, this situation is less than ideal. In contrast, when using the mutated L version, no differences in plasmid-based gene expression (i.e. Firefly controls) are observed, while genome replication and transcription are completely abolished. Thus, this mutant represents a superior control compared to the -L control, particularly in context of high-throughput assays. This is of particular importance as high throughput-screens under BSL4 conditions, which are necessary for work with infectious EBOV, are significantly more complex and cost-intensive than similar screens under BSL2-conditions, providing a strong incentive for the use of EBOV minigenome and other life cycle modelling systems for drug screening purposes [30]. Further, the development and use of similar catalytically inactive polymerase mutants in the place of conventional -L controls may represent a technical improvement for other minigenome systems (e.g. for other viruses) that may demonstrate more modest dynamic ranges and thus be more significantly impacted by such effects.

Overall, we have experimentally confirmed the catalytic center of the RdRp of the EBOV polymerase, which represents a promising target for the development of antivirals. This work provides a basis for future studies aimed at inhibiting the activity of this protein, which is absolutely crucial for the virus life cycle, as well as providing technical advancements in the tools available for high-throughput screening applications.
